# Correlation between S-1 treatment outcome and expression of biomarkers for refractory thymic carcinoma

**DOI:** 10.1186/s12885-016-2159-7

**Published:** 2016-02-25

**Authors:** Yusuke Okuma, Yukio Hosomi, Shingo Miyamoto, Masahiko Shibuya, Tatsuru Okamura, Tsunekazu Hishima

**Affiliations:** Department of Thoracic Oncology and Respiratory Medicine, Tokyo Metropolitan Cancer and Infectious diseases Center Komagome Hospital, 3-18-22 Honkomagome, Bunkyo, Tokyo 113-8677 Japan; Division of Oncology, Research Center for Medical Sciences, The Jikei University School of Medicine, Minato, Tokyo Japan; Department of Clinical Oncology, Japan Red Cross Medical Center, Shibuya, Tokyo Japan; Department of Pathology, Tokyo Metropolitan Cancer and Infectious diseases Center Komagome Hospital, Bunkyo, Tokyo Japan

**Keywords:** Thymic carcinoma, S-1, Rare cancer, Thymidine synthase, Orotate phosphoribosyltransferase, Dihydropyrimidine dehydrogenase

## Abstract

**Background:**

Thymic carcinoma is a rare cancer with minimal evidence of a survival benefit following chemotherapy. An oral fluoropyrimidine of S-1, however, is the recommended active cytotoxic chemotherapy agent for refractory thymic carcinoma based on a case series, whereas sunitinib or everolimus are recommended as molecular-targeted agents based on Phase II trials. We retrospectively investigated the efficacy of S-1 for refractory thymic carcinoma and performed a biomarker analysis.

**Methods:**

We assessed the clinicopathological variables of 14 consecutive patients who underwent S-1 for refractory thymic carcinoma and correlated the clinical outcomes with potential biomarkers using paraffin-embedded cancer tissues of eight patients in the cohort.

**Results:**

A total of 178 thymic malignancies were identified, of whom 14 patients included 12 cases of squamous cell carcinoma, one lymphoepithelioma-like carcinoma, and one undifferentiated carcinoma. Six patients exhibited a partial response (42.9 %: 95 % confidence interval [CI], 21.4–67.4) and the disease control rate was 85.7 % (60.0–96.0 %). After a median follow-up of 24.2 months, the median progression-free survival was 8.1 months (range, 2.6–12.2 months), and median overall survival was 30.0 months (range, 6.2–41.9 months). No significant correlation between biomarker expression and response was noted. However, thymidine synthase (TS)/dihydropyrimidine dehydrogenase and TS/orotate phosphoribosyltransferase were observed.

**Conclusions:**

S-1 for refractory thymic carcinoma offered clinical activity and achieved an 85 % disease control rate. Although the biomarkers did not correlate with clinical outcome, the study results showed efficacy of S-1 as a cytotoxic chemotherapy for refractory thymic carcinoma, which warrants future investigation.

**Electronic supplementary material:**

The online version of this article (doi:10.1186/s12885-016-2159-7) contains supplementary material, which is available to authorized users.

## Background

Rare cancers experience the crucial problem of slow in improvements of treatment and guideline because of the unfeasibility of large clinical trials. Also, a concise pathological diagnosis is often difficult. Recently, the RARECARE project [1] supported by the European Commission, focus on rare cancers to overcome these issues because they collectively represent about 22 % of all cancer cases despite the rarity of each of the individual 186 rare cancers [2]. According to the RARECARE definition, rare cancers have an incidence of less than 6 per 100,000 persons per year [3]. In addition, Rare Cancers Europe recently published a consensus position paper for clinical trials in rare cancers [4].

Thymic malignancies comprising of thymoma and thymic carcinomas are rare cancers according to the above definition. Thymic carcinoma represents a rare and aggressive histological subtype of thymic epithelial tumors with the absence of thymic function, whereas thymomas often have immunological complications. An annual incidence of 0.15–0.32 per 100,000 person-years in the United States and the Netherland is reported [5, 6]. Moreover, advanced stages at initial diagnosis are demonstrated in thymic carcinoma with metastasis or extension to surrounding tissues, whereas immune-active complications appears at early stages in thymoma. Thymomas can be classified into five groups: A, AB, B1, B2, and B3. Retrospective studies have shown types A and AB to have better prognoses than B1, B2, B3, and carcinomas, with thymic carcinoma in particular having a poor prognosis compared with thymomas, with a 5-year survival rate of 30–50 %. [7–9] The Masaoka–Koga staging system is widely accepted for both thymomas and thymic carcinomas, but this can lead to incorrect diagnoses, confusion between clinical entities, and mixing of management strategies. Treatment includes surgery for thymic carcinoma. However, patients with metastatic thymic carcinoma are treated with palliate-intent chemotherapy or best supportive care. Nevertheless, optimal chemotherapy has not been determined because of the rarity. Platinum combination chemotherapy with or without anthracycline is a widely used chemotherapy for thymic carcinoma as first-line chemotherapy, with response rates ranging from 20–50 % [10, 11].

There is no known survival benefit to second-line chemotherapy for refractory thymic carcinoma. According to the National Comprehensive Cancer Network (NCCN) guidelines for thymomas and thymic carcinomas, for patients with recurrent thymoma and thymic carcinoma, the recommended chemotherapy is single-agent or clinical trial [12]. Recent results of most molecular targeted agents are disappointing; however, sunitinib [13] and erverolimus [14] showed verified activities in a phase II study. Our institution reported that cytotoxic chemotherapy is anticipated for refractory thymic carcinoma based on retrospective studies [15]. We have already reported the clinical response of a small case series to S-1 in four patients with refractory thymic carcinoma, which was cited in the NCCN guidelines [16].

Many metabolic enzymes play key roles in the metabolism of fluoropyrimidines, including S-1. Among these, thymidine synthase (TS) is the rate-limiting enzyme in the *de novo* synthesis of 2′-deoxy-thymidine-5′-monophosphate, which is necessary for DNA synthesis and repair, and is therefore the primary target of fluoropyrimidines [17]. Dihydropyrimidine dehydrogenase (DPD) is the rate-limiting enzyme in 5-fluorouracil (5-FU) catabolism [18], while thymidine phosphorylase (TP) and orotate phosphoribosyltransferase (OPRT) convert 5-FU to active metabolites such as 2′-deoxy-5-fluorouridine and 5-fluorouridine-5′-monophosphate, respectively. Previous studies found that increased expression levels of TS, DPD, and TP, and reduced expression of OPRT in tumors, were associated with low sensitivity to fluoropyrimidine-based chemotherapy [17–20]. However, the correlation between clinical outcome and the expression of these genes remains unclear. Furthermore, thymic carcinomas show low expression of TS, suggesting the potential therapeutic value of TS-targeted agents including fluoropyrimidine or antifolate agents [21]. However, no studies have focused on chemotherapy in relation to TS expression in thymic carcinomas.

In this investigation, we retrospectively evaluated the clinical outcomes of S-1 treatment in 14 consecutive patients with refractory thymic carcinoma with progressive disease after at least one prior session of platinum-based chemotherapy. We also analyzed the mRNA expression levels of TS, DPD, OPRT, and TP to identify potential biomarkers of S-1 response.

## Methods

### Database for clinical data and acquisition of cancer specimens

Among total of 178 thymic malignancies (100 thymoma and 78 thymic carcinoma) were identified, a series of 13 patients with thymic carcinoma was treated at Tokyo Metropolitan Cancer and Infectious diseases Center Komagome Hospital (Tokyo, Japan) among April 1987 and December 31, 2014. Also, one patient at Japan Red Cross Medical Center (Tokyo Japan), for a total of 14 consecutive patients. All patients had histologically confirmed metastatic or relapsed thymic carcinoma not amenable to curative-intent treatment and disease progression after failure of at least one previous treatment with platinum-based chemotherapy. The diagnosis was confirmed with immunohistochemistry using CD5 and/or c-Kit to exclude other thoracic malignancies, and terminal deoxynucleotidyl transferase (TdT) to distinguish from thymoma. We evaluated laboratory data from all patients, and targeted lesion responses were determined with computed tomography. All patients who were treated with chemotherapy gave informed consent.

In the present analysis, we collected and assessed treatment outcomes according to the International Thymic Malignancies Interest Group (ITMIG) Policies and Management [22]. The medical records and laboratory data of each patient were retrieved for analysis and assessment according to treatments for thymic carcinoma, and associated complications were also reviewed. Data on significant hematological and non-hematological toxicities associated with chemotherapy were also collected.

The baseline demographic characteristics are summarized in Table 1. The median age of all patients was 56 years (range, 14 to 81 years). On histology, there were three subtypes of thymic carcinoma: squamous cell carcinoma (SQC), undifferentiated carcinoma, and lymphoepithelioma-like carcinoma. No cases of myasthenia gravis or other associated symptomatic paraneoplastic syndromes were observed.

**Table 1:** Demographic and baseline patient characteristics

Characteristics	No of patients (*n* = 14)	Percent
Gender		
Male	6	42.9
Female	8	57.1
Age, median (range)/years	56.5 (14-81)	
Performance status (ECOG)		
0–1	13	92.9
2	1	7.1
Stage at diagnosis (Masaoka-Koga)		
IVa	3	21.4
IVb	8	57.1
Recurrence	3	21.4
Metastatic sites (overlapped)		
Lung	6	
Liver	2	
Lymph nodes	5	
Pleura	5	
Bone	3	
Brain	1	
Histologic subtype		
Squamous cell carcinoma	12	85.7
Lymphoepithelioma-like carcinoma	1	7.1
Undifferentiated carcinoma	1	7.1
Previous chemotherapy		
ADOC	3	
Cisplatin-irinotecan	9	
Cisplatin-gemcitabine	1	
Carboplatin-gemcitabine	1	

*No* number, *ADOC* cisplatin, adriamycin, vincristine, and cyclophosphamide

### Treatment with S-1

All patients had been previously treated with fluoropyrimidine-agent besides those previously treated with platinum-based chemotherapy as a front-line approach. Also, all of the 14 patients that were administrated S-1 had an Eastern Cooperative Oncology Group (ECOG) performance status (PS) of less than 2 with reserve adequate bone marrow. Also, physicians were complied with the assurances of safety provided to the Taiho Pharmaceutical Co. to promise safety. The initial dose of S-1 was determined based on body surface area (BSA): 80 mg daily for BSA <1.25 m^2^; 100 mg daily for 1.25 < BSA <1.5 m^2^; and 120 mg daily for BSA ≥1.5 m^2^. The drug was taken twice daily for 4 weeks, followed by 2 weeks-off, which comprised a cycle. In cases of poor PS, mild organ impairment, elderly patients, or other reasons suggesting intolerability, the dose was decreased stepwise or given for 2 weeks-on and followed by a 1-week drug-free interval per cycle. If a dose reduction of 20 % was required, the patients continued to receive the reduced dose throughout their treatment.

### Assessment and outcomes

The benefits of treatment were retrospectively evaluated using the following: response rate, disease control rate, progression-free survival (PFS), overall survival (OS), and 1-year survival rate. We assessed treatment efficacy of S-1 using the Response Evaluation Criteria in Solid Tumors criteria version 1.1 (RECIST). Patients were assessed at least every 2 months by CT. S-1 treatment was reported from the date of the first cycle to confirm of disease progression. Disease control rate was defined as objective responder plus stable disease. PFS was calculated from the first date of S-1 until the date of confirmed progression, early discontinuation of treatment, or death from any cause and was censored at the date of the last follow-up visit for patients who were still alive and who had not progressed. OS was defined as the interval between the first date of treatment to the time of death from any cause or the last follow-up evaluation. Patients who were alive on the date of last follow-up were censored on that date. Because of the retrospective nature of the data, these relevant end points were chosen to reflect clinical practice.

Hematological and non-hematological toxicities related to chemotherapy were described using the common toxicity criteria according to the Common Terminology Criteria for Adverse Events version 4.0 (CTCAE v4.0) from medical records.

### Tumor quantitative assessment for reverse transcription-PCR

In the available cohort, eight specimens that were treated with S-1 and capecitabine, a similar fluoropyrimidine agent, were evaluated for potential biomarkers with formalin-fixed paraffin-embedded (FFPE) primary thymic carcinoma specimens (5 core needle biopsy, 2 surgical specimens, and 1 excision biopsy) at diagnosis using PCR quantification of mRNA expression of TS, DPD, OPRT, and TP. In the other five patients, sufficient specimens were not obtained for analysis because of core-needle biopsies or decalcified processes with bone metastasis.

Representative hematoxylin and eosin-stained slides from FFPE specimens were reviewed by a pathologist for a manual macrodissection of tumor tissue. Tumor tissue was selected and dissected using a scalpel. RNA was isolated from tumor tissue using RNeasy FFPE Kit (Qiagen, Chatsworth, GA, USA). cDNA was prepared using High Capacity Reverse Transcription Kit (Life Technologies, Foster City, CA, USA) according to the manufacturer’s instructions.

The expression levels of four genes were determined using TaqMan real-time PCR (TaqMan array card; Life Technologies) after TaqMan assay-based pre-amplification. Briefly, cDNA (2.5 μL) was pre-amplified using TaqMan PreAmp Master Mix (2×; Life Technologies) and a pool of TaqMan® Gene Expression Assays (0.2×) in a 10-μL PCR reaction. The pre-amplification cycling conditions were as follows: 95 °C for 10 min followed by 14 cycles of 95 °C for 15 s and 60 °C for 4 min. An amplified cDNA sample was diluted 20 times in TE Buffer. Next, 25 uL of amplified cDNA was added to 25 μL of RNase-free water and 50 μL of 2× TaqMan Gene Expression Master Mix (Life Technologies). The mixture was then transferred into a loading port for the TaqMan array card. The card was centrifuged twice, sealed, and PCR amplification was performed using the Applied Biosystems Prism 7900HT Sequence Detection System (Life Technologies) under the following thermal cycling conditions: 50 °C for 2 min and 94.5 °C for 10 min followed by 40 cycles of 97 °C for 30 s and 59.7 °C for 1 min. The array card included ACTB, GAPDH, and RPLP0 as references based on their proven roles as housekeeping genes [23, 24]. The assay IDs used in the array card are shown in Additional file 1: Table S1. The cycle threshold (Ct) value, which is inversely proportional to the amount of cDNA, was calculated. The gene-expression (relative mRNA) levels were expressed as the ratio (the differences between the Ct values) between the gene of interest and the geometric mean of the reference genes, which provided a baseline measurement for the amount of mRNA isolated from a specimen.

### Statistical methods

PFS and OS were estimated according to the Kaplan–Meier method. Differences between tumor response of S-1 and the mRNA expression of TS, TP, DPD, or OPRT were evaluated with the Wilcoxon rank sum test. We also analyzed four biomarkers and responders with a correlation coefficient matrix. All statistical analyses were carried out using JMP 11 (SAS Institute Inc., Cary, NC, USA). A *P*-value less than 0.05 was considered significant. This retrospective study was approved by the institutional review board (IRB) of Tokyo Metropolitan Cancer and Infectious diseases Center Komagome Hospital (#1049). Written informed consent was obtained from each patient before use of S-1 for advanced thymic carcinoma in the clinical setting, however, the IRB waived the requirement for obtaining informed consent to biomarker analysis with tumor specimens.

## Results

### Treatment efficacy

**Fig. 1:**
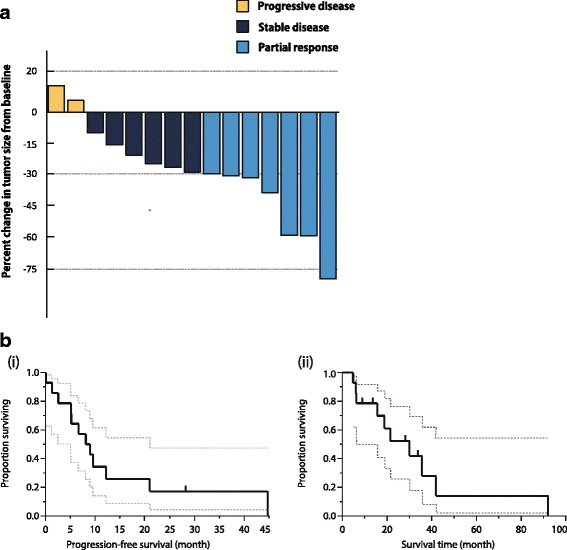
Clinical outcome of S-1 for relapsed thymic carcinoma. **a** Best response of targeted lesions to S-1 treatment. **b** (i) and (ii) Kaplan–Meier analyses for PFS and OS of S-1 in refractory thymic carcinoma

Disease control was observed in 12 patients (85.7 %), with 6 partial responses (42.9 %) recorded. There were no complete responders (Fig. 1a). The median PFS was 8.1 months (95 % confidence interval [CI], 2.6–12.2 months), while the median OS was 30.0 months (95 % CI, 6.2–41.9 months) after a median follow-up of 24.2 months. PFS and OS curves are shown in Fig. 1b, and effectiveness is summarized in Table 2. The 1-year survival rates were 68.8 %. Previous lines of chemotherapy included an average of two lines.

**Table 2:** Clinical outcome of S-1 treatment for refractory thymic carcinoma

Clinical outcome	N° of patients (*n* = 14)	
Response to chemotherapy	N (%)	95 % CI
Complete response	0 (0 %)	
Partial response	6 (42.9 %)	[21.4–67.4]
Stable disease	6 (42.9 %)	[21.4–67.4]
Progression disease	2 (14.3 %)	[0.04–39.9]
Median response duration, moths [95 % CI]	8.1	[2.6–12.2]
Median overall survival, months [95 % CI]	30.0	[6.2–41.9]
1-year survival rate, %	68.8	

*CI* confidence interval

Grade 3 and 4 treatment related toxicities in CTCAE was seen in 21.4 % of patients. Among them, six patients needed a dose reduction because of toxicities and treatment was discontinued in four patients before progression. In these patients, treatment was stopped early to preserve the benefits achieved. Two patients requested that treatment be stopped due to anorexia. There were no toxic deaths.

### TS, DPD, OPRT, and TP mRNA expression

**Fig. 2:**
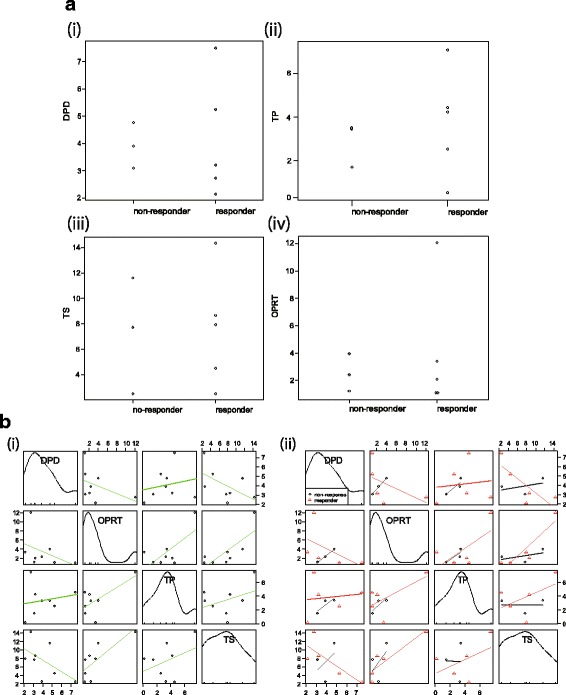
Correlation to expression of TP, DPD, OPRT, and TP in tumour specimens (**a**) Relationship between biomarkers and tumour response. (i) DPD, (ii) TP, (iii) TS, and (iv) OPRT. **b** Correlation between biomarkers. **b** (i) DPD, TP, TS, and OPRT; (ii) correlation between responders and non-responders

Histograms of the expression values for each gene are shown in Fig. 2a. Each relative mRNA level at their respective 95 % CIs was as follows: TS relative mRNA expression levels had median values of 7.81 (2.44–14.30), DPD 3.56 (2.14–7.49), OPRT 2.24 (1.06–12.04), and TP 3.36 (0.22–7.48). A correlation between the expression of TS, DPD, TP, and OPRT genes on survival in this cohort was not seen. There was a significant association between TS and DPD or TS and OPRT mRNA expressions in each group (Fig. 2b).

## Discussion

The present retrospective study demonstrated the potential activity of S-1 in 14 patients with refractory thymic carcinoma. However, a significant correlation between S-1 response and hypothesized biomarkers was not seen.

Platinum-combination chemotherapy plays an important role in the first-line chemotherapy for advanced thymoma and thymic carcinoma [11]. However, the rarity of this disease has hampered the development of an optimal chemotherapy regimen. The clinical benefit of chemotherapy remains uncertain compared with the best supportive care for thymoma and thymic malignancies. For refractory thymoma and thymic carcinoma, the NCCN guidelines [12] recommend single-agent or non-platinum-based chemotherapy, such as etoposide [25], gemcitabine [26], paclitaxel [27], ifosfamide [28], pemetrexed [29], 5-fluorouracil (5-FU) and leucovorin [30], and octreotide (including long acting-formulation) plus prednisolone [31]. The NCCN guidelines do not provide separate recommendations for thymic carcinoma and thymoma [32]. Amrubicin [33], docetaxel [34], and S-1 [16, 35, 36] were reported to be effective for thymic carcinoma. Recently, molecular investigations and clinical outcomes of thymic malignancies have demonstrated that thymoma and thymic carcinoma are separate diseases and classified according to the World Health Organization 2004 [37]. Therefore, the ITMIG recommends further investigation to differentiate these cancers as two separate categories and to not combine them as was the case in the past. A European collaborative investigation also demonstrated that the prognosis for thymic carcinoma in children was poor compared with that for thymoma [38]. Molecular targeted agents are currently being investigated. Based on c-Kit gene mutant status, drugs that inhibit c-Kit, such as imatinib [39], sorafenib [40], and sunitinib [41], may provide better results [42]. Recent trials with molecular targeted drugs did not meet expectations (Table 3). However, sunitinib, and everolimus for refractory thymic carcinoma, and cixutumumab for refractory thymoma have an anticipated clinical benefit in phase II studies. If limited to case series, cytotoxic chemotherapy demonstrates clinical activity for refractory thymic carcinoma [15]. The endpoints of time-to-event have influence on the selection bias easier than response rate except for the efficacy of chemotherapy, such as the progress of supportive care, therefore, suggesting that response rate is a suitable endpoint for comparing chemotherapeutic regimens.

**Table 3:** Chemotherapy and molecular-targeted agents for refractory thymic carcinoma

Authors	Agent	Study design	Target	Number	Response rate (DCR)	PFS (month)	OS (month)
Cytotoxic agents							
Loehrer et al. [29]	Pemetrexed	Ph II	-	11	NR	1.3	N/A
Wakelee et al. [33]	Amrubicin	Ph II	-	19	10.5 %	8.5	18.1
Liang et al. [46]	Pemetrexed	Retrosp	-	10	10.0 %	6.5	12.7
Palmieri et al. [47]	Capecitabine + gemcitabine	Ph II	-	8	37.5 %	6 (3–10)	N/A
The present study	S-1	Retrosp	-	14	42.9 %	8.1	30.0
Molecular targeted agents							
Thomas et al. [13]	Sunitinib	Ph II	c-KIT, PDGFR	23	26 % (65 %)	7.2	Not reached
Zucali et al. [14]	Everolimus	Ph II	mTOR	12	25 % (41 %)	12.1^a^	24.0^a^
Rajan et al. [48]	Cixutumumab	Ph II	IGF-1R	12	0 %	1.7	8.4
Giaccone et al. [49]	Belinostat	Ph II	HDAC	16	0 % (50 %)	5.8	12.4
Besse et al. [50]	Milciclib (PHA-848125 AC)	Ph II	CDK, src family	26	-	-	-
Bedano et al. [51]	Erlotinib + bevacizumab	Ph II	EGFR, VEGF	7	0	N/A	N/A
Kurup et al. [52]	Gefitinib	Ph II	EGFR	7	0	N/A	N/A
Giaccone et al. [53]	Imatinib	Ph II	c-KIT mutation	5	0	N/A	N/A
Loehrer et al. [54]	Octreotide + prednisone	Ph II	somatostatin receptor	6	0	4.5	23.4
Gubens et al. [55]	Saracatinib (AZD0530)	Ph II	src family	9	0	3.6	6.7

*n* number, *PFS* progression-free survival, *DCR* disease control rate, *OS* overall survival, *Ph II* phase II, *Retrosp* retrospective, *IGF-1R* insulin-like growth factor 1 receptor, *HDAC* histone deacetylase, *PDGFR* platelet-derived growth factor, *CDK* cyclin-dependent kinase, *mTOR* mammalian target of rapamycin. *EGFR* epidermal growth factor, *VEGF* vascular endothelial growth factor

^a^Survival data include thymoma and thymic carcinoma

S-1 (TS-1; Taiho Pharmaceutical Co., Ltd, Tokyo, Japan) is an oral fluoropyrimidine agent composed of tegafur, 5-chrolo-2, 4-dihydroxypyridine (gimeracil: CDHP), and oteracil potassium (oxonic acid: Oxo) in a molar ratio of 1:0.4:1. TS, a critical source of thymidine nucleotides for DNA synthesis and repair, is the target enzyme for 5-FU metabolite and 5-fluoro-deoxyuridinemonophosphate (FdUMP). Several studies have demonstrated that high expression of TS level correlates with 5-FU resistance in various cancers. 5-FU is then degraded by DPD, which inhibits TS. CDHP competitively inhibits DPD, which is involved in the degradation of 5-FU, thereby increasing serum 5-FU concentrations. It is expected to act more intensively than older fluoropyrimidine agents, but increased 5-FU concentrations in the intestinal mucosa leads to severe gastrointestinal toxicities. Oxo inhibits diarrhea by selective inhibition of OPRT and palliates diarrhea. Therefore, intensive treatment with S-1 is available with Oxo to reduce GI toxicity. Currently, S-1 has demonstrated activity against a broad spectrum of solid tumors, such as gastric cancer, head and neck cancer, non-small cell lung cancer, pancreatic cancer, bile duct cancer, breast cancer, and colon cancer.

The response rate of the present study was 42.9 % with a 95 % CI of 21.4–67.4 and a median progression-free survival of 8.1 months (2.6–12.2). Sunitinib is a promising agent with the biological plausibility of inhibition for c-Kit and antiangiogenic effects [41]. Everolimus was tested for thymic malignancies in a phase I study [14] and inhibition of a common cancer signaling pathway, serine–threonine kinase mammalian target of rapamycin (mTOR), is proposed. However, our results did not provide the promising biological plausibility of S-1 for thymic carcinoma. On the other hand, the cost-benefit is also different among these agents and is of interest. Calculating from the results of median PFS from phase II trials of sunitinib, everolimus, and the present retrospective study of S-1 for thymic carcinoma, a total of 7.2 months of sunitinib treatment costs about 30,000 EUR (5,700 EUR per each cycle (6 weeks)), and a total of 12.1 months of everolimus treatment costs 68,500 EUR (7,300 EUR per 6 weeks), and a total of 8.1 months of S-1 treatment costs 4,200 EUR (700 EUR per cycle (6 weeks)). With a cost–benefit balance, cytotoxic agents should progress in clinical trials or observational studies because response rates even in second-, third-, and fourth-lines of cytotoxic chemotherapy in our result decreased to 39.1 %, 23.1 %, and 12.5 %, respectively [15]. As for suggested biomarkers for S-1, thymic carcinoma showed significantly progressively decreased levels of TS mRNA expression from type B1 to carcinoma. Kaira et al. demonstrated that TS was potentially correlated with TS in thymic carcinoma using immunohistochemistry staining [43]. Therefore, the possible biological plausibility for S-1 activity in thymic carcinoma was suggested. However, the present study did not show a significant relationship between response and expression of biomarkers. However, a correlation between TS/DPD and TS/OPRT may potentially be seen in the Fig. 2. Unfortunately, the results are inconclusive because of small sample size and the number of specimens analyzed at diagnosis. TS, OPRT, and DPD are definitive biomarkers for fluoropyrimidine is not always active with a biological plausibility in thymic carcinoma. Also, TP was not unclear to anticipate to be active of capecitabine.

The key limitation of the present study was the small number of patients and the lack of accurate clinical confirmation of response and time-to-events because of its retrospective nature. However, this is a common limitation of retrospective studies of rare cancers. Second, the methodology of analysis for TS, TP, OPRT, and DPD mRNA expression were examined in the small number of specimens at initial diagnosis. Thus, histological features or characteristics might have been modified by prior chemotherapy. The main reason for the unavailability of specimens for biomarker analysis was scant tissue from core needle biopsy for advanced stage, whereas sufficient specimen volumes were obtained for recurrent cases.

This results of the present study support the multicenter, uncontrolled, open-label phase II trial to confirm our findings at three cancer centers in Tokyo (National Cancer Center Hospital, The Cancer Institute Hospital of the Japanese Foundation for Cancer Research, and Tokyo Metropolitan Cancer and Infectious diseases Center Komagome Hospital) that were launched (UMIN000010736) with a central pathological diagnosis and referring to the position paper in rare cancer trial [4]. However, clinical evidence for rare cancers is challenging and hampers enrolling patients. The enriched expression of PD-L1 in thymic carcinoma cancer cells suggests that immune-checkpoint inhibitors may represent an interesting new therapeutic modality for thymic carcinomas (NCT02364076) [44, 45]. However, suitable biomarkers for immune-checkpoint inhibitors remain uncertain, and cytotoxic chemotherapy for relapsed thymic carcinoma should thus be developed simultaneously.

## Conclusions

The present retrospective analysis of 14 patients with refractory thymic carcinoma who were treated with single agent S-1 demonstrated clinical activity. Further clinical management strategies and treatments for refractory thymic carcinoma should be investigated.

## Additional file

Additional file 1: Table S1. Clinical characteristics of patients treated with S-1 for refractory thymic carcinoma. (DOCX 18 kb)

## Abbreviations

5-FU: 5-fluorouracil
BSA: body surface area
CTCAE: Common Terminology Criteria for Adverse Events
DPD: dihydropyrimidine dehydrogenase
ECOG: Eastern Cooperative Oncology Group
FdUMP: 5-fluoro-deoxyuridinemonophosphate
FFPE: formalin-fixed paraffin-embedded
ITMIG: International Thymic Malignancies Interest Group
NCCN: National Comprehensive Cancer Network
OPRT: orotate phosphoribosyltransferase
OS: overall survival
PFS: progression-free survival
PS: performance status
RECIST: Response Evaluation Criteria in Solid Tumors criteria
SQC: squamous cell carcinoma
TdT: terminal deoxynucleotidyl transferase
TP: thymidine phosphorylase
TS: thymidine synthase
