# Erratum to: Correlation between S-1 treatment outcome and expression of biomarkers for refractory thymic carcinoma

**DOI:** 10.1186/s12885-016-2309-y

**Published:** 2016-04-15

**Authors:** Yusuke Okuma, Yukio Hosomi, Shingo Miyamoto, Masahiko Shibuya, Tatsuru Okamura, Tsunekazu Hishima

**Affiliations:** Department of Thoracic Oncology and Respiratory Medicine, Tokyo Metropolitan Cancer and Infectious diseases Center Komagome Hospital, 3-18-22 Honkomagome, Bunkyo, Tokyo, 113-8677 Japan; Division of Oncology, Research Center for Medical Sciences, The Jikei University School of Medicine, Minato, Tokyo, Japan; Department of Clinical Oncology, Japan Red Cross Medical Center, Shibuya, Tokyo, Japan; Department of Pathology, Tokyo Metropolitan Cancer and Infectious diseases Center Komagome Hospital, Bunkyo, Tokyo, Japan

## Erratum

Following publication of this work [1] the authors noticed a mistake within the article’s competing interests section. The correct competing interests section is provided below.
