# Supplementary material for: Correlation between S-1 treatment outcome and expression of biomarkers for refractory thymic carcinoma
Source: BMC Cancer. 2016 Feb 25;16:156. doi: 10.1186/s12885-016-2159-7 (PMC4766615; doi:10.1186/s12885-016-2159-7)
Supplement: Additional file 1: Table S1. — Clinical characteristics of patients treated with S-1 for refractory thymic carcinoma. (DOCX 18 kb) [file 12885_2016_2159_MOESM1_ESM.docx]

| No. | Subtype | Metastasis | First-line  chemotherapy | treatment lines  of S-1 | No of cycles  of S-1 | Response in RECIST criteria | PFS  (mo) | OS  (mo) |
| --- | --- | --- | --- | --- | --- | --- | --- | --- |
| 1 | SQC | HEP, OSS | IP | 3 | 2 | PD | 2.6 | 4.8 |
| 2 | SQC | PUL, BRA | IP | 5 | 5 | SD | 8.1 | 35.7 |
| 3 | SQC | PLE | IP | 4 | 8 | SD | 12.2 | 15.6 |
| 4 | LELC | OSS | ADOC | 2 | 1 | PD | 1.4 | 6.1 |
| 5 | SQC | PLE | IP | 4 | 1 | SD | 0.1 | 30.0 |
| 6 | undiff | OSS | IP | 2 | 2 | PR | 9.0 | 21.4 |
| 7 | SQC | PUL, LYM | IP | 3 | 3 | SD | 5.1 | 6.2 |
| 8 | SQC | PLE, PUL, LYM | IP | 3 | 14 | PR | 21.0 | 33.8 |
| 9 | SQC | HEP, PUL | CG | 2 | 4 | PR | 6.7 | 18.8 |
| 10 | SQC | PUL, OSS | CbG | 2 | 4 | SD | 5.2 | 41.9 |
| 11 | SQC | PUL, PLE | ADOC | 2 | 30 | PR | 44.8 | 92.1 |
| 12 | SQC | LYM, PLE | IP | 2 | 6 | PR | 9.6 | 13.6 |
| 13 | SQC | LYM | ADOC | 2 | 19 | PR | 28.3 | 28.3 |
| 14 | SQC | PLE, LYM | IP | 2 | 6 | SD | 9.0 | 9.0 |

**Supplementary Table** Clinical characteristics of patients treated with S-1 for refractory thymic carcinoma

SQC, squamous cell carcinoma; undiff, undifferentiated carcinoma; HEP, liver; OSS, bone; PUL, lung; BRA, brain; PLE, pleura; LYM; lymph node; IP, irinotecan+cisplatin; ADOC, cisplatin+adriamycin+vincristine+cyclophosphamide; CG, cisplatin+gemcitabine; CbG, carboplatin+gemcitabine; PR, partial response; SD, stable disease; PD, progressive disease; PFS, progression-free survival; OS, overall survival; mo, months.
